# A Low-Cost Multi-Purpose IoT Sensor for Biologging and Soundscape Activities

**DOI:** 10.3390/s22197100

**Published:** 2022-09-20

**Authors:** Dinarte Vasconcelos, Nuno Jardim Nunes

**Affiliations:** ITI/LARSyS, Instituto Superior Técnico, Av. Rovisco Pais 1, 1049-001 Lisbon, Portugal

**Keywords:** multipurpose sensor, biodiversity monitoring, optical detection, acoustic capability, LoRa technology, embedded systems

## Abstract

The rapid expansion in miniaturization, usability, energy efficiency, and affordability of Internet of Things (IoT) sensors, integrated with innovations in smart capability, is greatly increasing opportunities in ground-level monitoring of ecosystems at a specific scale using sensor grids. Surrounding sound is a powerful data source for investigating urban and non-urban ecosystem health, and researchers commonly use robust but expensive passive sensors as monitoring equipment to capture it. This paper comprehensively describes the hardware behind our low-cost, small multipurpose prototype, capable of monitoring different environments (e.g., remote locations) with onboard processing power. The device consists of a printed circuit board, microprocessor, local memory, environmental sensor, microphones, optical sensors and LoRa (Long Range) communication systems. The device was successfully used in different use cases, from monitoring mosquitoes enhanced with optical sensors to ocean activities using a hydrophone.

## 1. Introduction

Real-time analysis of data from recording devices is increasingly becoming a branch of ecological research, with multiple research projects using low-cost microcontrollers and other expensive programmable devices [1] to broadcast the environmental data for analysis. This approach has found a vast range of applications, from monitoring traffic noise in the cities [2] to autonomous recording devices widely used in bird species [3].

Due to these environmental acoustic devices’ cost and energy efficiency, the processing is now carried out on the microcontroller to detect species presence from an ecosystem of interest through detection algorithms to recognize the unique vocalizations and infer the species richness. Still, challenges remain in estimating the population densities through the number of species.

While all organisms in the ecosystem are vital, the role played by insects is especially critical. They create the biological foundation for all terrestrial ecosystems, forming the basal part of the food pyramid and influencing our agriculture ecosystems and human health [4]. They cycle nutrients, pollinate plants [5,6], disperse seeds, preserve soil structure and fertility, control populations of other organisms, and supply a significant food source for other species [7,8] and show how these relate to the environment conditions [9]. Insects also transmit infectious pathogens (parasites, viruses and bacteria) between humans or from animals to humans. Every year, more than 700,000 people die from diseases such as malaria and dengue [10]. However, many more vector-borne conditions cause chronic suffering, life-long morbidity and disabilities, disproportionately affecting the poorest populations in tropical and subtropical areas [11].

The use of acoustic sensors to monitor insects is a significant trend in recent research, monitoring and ecological practice. However, some limitations with acoustics have arisen due to the emergence of optical strategies to observe flying insects based on light scattering with a fast read-out ability to guarantee the capturing of wing beats [12,13]. This technique is efficient in saving battery life and an accurate way to estimate the population densities of the insects’ species. Furthermore, these self-made devices are also improved with current machine learning, deep learning, and general-purpose detection algorithms. These let us achieve a very high improvement rate in remote monitoring data, with no need for manual recalibration and pretraining of the device for the target phenomena or the acoustic requirements in the field.

In addition, to further increase the durability of dedicated, power-efficient acoustic sensors, the devices are set to record in response to specific sounds triggered by the detection algorithms instead of continuous recording at regular intervals. This significantly reduces energy consumption because writing an audio file on the device is one of the most power-consuming tasks. Moreover, this reduces the storage requirements and battery costs, resulting in a lower price for long-term monitoring systems.

In this paper, we propose and describe a low-cost, multipurpose device to monitor and control insect/bird densities, where the scientific community can take measures either to eradicate the most harmful species for human health or to preserve essential species in the ecosystem. The optical-acoustic device described here is more comprehensive and robust than available passive acoustic monitoring devices nowadays. Our sensor provides considerably greater portability, ease of use, and scale for rural and urban locations with LoRa and high durability in the field with smaller batteries and solar panels. The sensor has the possibility for various applications in large-scale, long-term surveillance (e.g., the possibility of using microphones, ultrasonic mics, and hydrophones) and optical detection. Continuing developments in intelligent, energy-efficient techniques and decreasing unit costs, we are working towards giving local communities the ability to afford to remotely survey their areas with a particular device rather than multiple devices for every task, helping to lower overall system costs for emerging countries.

The remainder of this paper is organized as follows: In Section 2, we briefly overview current devices available in the market. Section 3 describes the construction of the low-cost multipurpose sensor, from the design to LoRa communication technology, passing by the optical-acoustic system and power management. In Section 4, we show the bill of materials and the cost of the device. Section 5 exposes how we can validate and use the device. Finally, Section 6 shows the typical applications of the sensor in different scenarios and the use of machine-learning techniques capable of running on our sensor.

## 2. Hardware Overview

The analysis of urban, rural, and environmental sounds is a rapidly growing branch of biodiversity research, frequently referred to as bioacoustics, ecoacoustics, and recently optoacoustic recordings [14,15,16]. The research relies on analyzing stored datasets of collected sound, contributing to the evolution of science and monitoring procedures. In addition, a growing number of studies are often collated from audio-visual acoustic recordings on mains-powered acoustic designs leading to a demand for battery-powered passive acoustic monitoring (PAM) devices [17] in the life sciences. An acoustic sensor can be associated with any combination of a sound detector, microphone, and hydrophone created to detect and record audio in the surrounding environment. The importance of such sensors and a complete list of existing hardware and open-source software tools for analysis over a wide range of spatiotemporal patterns acoustic wildlife monitoring data can be found in the WWF guidelines for passive bioacoustic monitoring in biodiversity and conservation [18]. Most ecological and conservation research projects traditionally use PAM through commercial devices, such as the SongMeter series from Wildlife Acoustics (www.wildlifeacoustics.com, accessed on 20 July 2022), aquatic SoundTrap from Ocean Instruments (www.oceaninstruments.co.nz, accessed on 20 July 2022) and the BAR series from Frontier Labs (www.frontierlabs.com.au, accessed on 20 July 2022). These standard portable devices are appreciated for their excellent recording quality, making them suitable for studying acoustically sensitive organisms. However, they are still a costly research tool for monitoring uses, ranging in price from USD 250 to thousands of dollars.

This cost restricts usage for many of the research projects requiring coverage of large areas. In specific applications, such as acoustics monitoring biodiversity, inexpensive devices allowing researchers to cover more extensive areas could have benefits and have an acceptable trade-off between audio quality and cost. For this purpose, suitable and customized solutions have rapidly increased over the last three years, usually facilitated by new low-cost technologies [19].

State-of-the-art dedicated-designed acoustic sensors nowadays are expensive, so significant initial expenses are associated with setting up an acoustic survey program. For citizen science, this subject is still an obstacle to the broader uptake of acoustic monitoring, especially for conservation programs with a limited budget. Although there are several promising techniques for the development of low-cost customizable acoustic sensors (e.g., AudioMoth [20] and Solo [21]) and the use of smartphones as acoustic sensors for citizen science [22]. Nevertheless, these low-cost bioacoustic sensors have great potential to involve the citizens in general to collect and implement ecological data in developing countries.

Ongoing maintenance and regular data recovery for the long-term development of these sensors are required. Therefore, a more significant effort is needed on the researcher’s part, and the cost of maintaining such data collection devices is higher, especially in remote environments (e.g., tropical forests) principality without communication technology. Another disadvantage of the devices enunciated above is that they do not allow you to hear the sounds captured on the ground to check their quality more quickly and adapt the quality parameters of the captured sounds, needing a PC to complete that task. In remote areas, having a device with a system capable of charging its battery is essential to guarantee the greater longevity of the device. None of the current devices has a self-charge system. The future is moving toward automated wireless networked devices, with data automatically sent to a base station, potentially decreasing such costs significantly.

In order to reduce the cost associated with the acoustic monitoring market, the hardware community launched accessible, affordable and very small computers entirely embedded in a PCB (e.g., Raspberry PI and Asus tinker board) [23,24,25]. However, despite reducing the unit cost of such build-it-yourself devices, they present higher consumption, as long-term surveillance systems require a higher capacity battery to sustain such survey programs. Another disadvantage is the knowledge of software and electronics required to build each sensor and apply them in the field—knowledge that biologists and experts in biology do not normally have.

In addition, a device with only the acoustic monitoring approach has several disadvantages. These include its inability to detect living beings that do not emit sound and estimate the species density when the ambient noise is higher. Furthermore, they depend on relatively expensive equipment and highly skilled assistance to analyze the often massive volumes of data, and most of the devices can not provide communication with each other.

Bearing these factors in mind, we built a multipurpose device capable of adapting to different ecological research requirements with a low production cost compared to commercial sensors available in the market. This portable prototype can run lite machine learning algorithms, as it can send data through Long Range (LoRa) technology. It can be used in remote locations, making it available almost in real-time. These devices can also be scalable, allowing a mesh communication system between them until reaching the main gateway.

## 3. System Design

**Figure 1:**
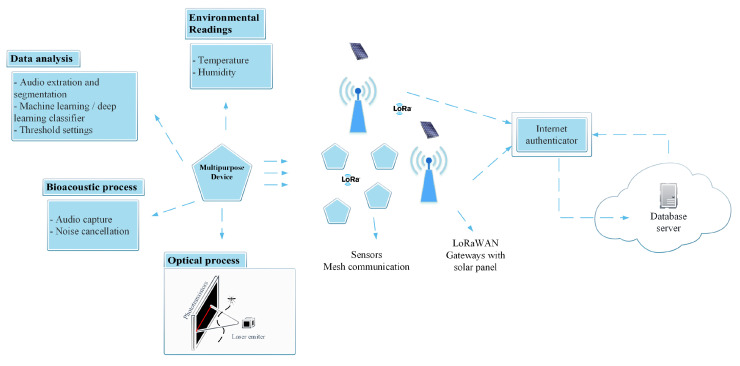
Multipurpose sensor overview.

Devices based on electro-acoustic transducer housing (MEMS) and application-specific integrated circuits (ASIC) in a single package allowed the development of cheaper, smaller, faster and energy-efficient ecoacoustic sensors. The optoacoustic sensor described here overcomes the barriers set by acoustic-only approaches based on MEMS. We propose a multipurpose device coupling acoustic sensor with optical sensors for inaudible insects and population estimation (e.g., pollinating insects and mosquitoes) jointly with the LoRa communication technology for efficient data transmission. Biodiversity monitoring in remote places is an essential topic but often problematic. Therefore, in this battery-powered board, we can also include a renewable power source (e.g., a solar panel) to improve the active time of the board. The optical process complements the acoustic sensing by detecting/counting small insects and activating the acoustic recording trigger when a flying insect passes in the IR detection field. The bioacoustic procedure identifies the species by utilizing audio features and machine learning/deep learning approaches. Through onboard processing algorithms, the data about the detected species is forwarded through a LoRa communication protocol until reaching the LoRa gateway or a mesh communication between the IoT devices until obtaining a base station. Then, in the server, all the statistics and predictions can be made and delivered to the responsible authorities. Figure 1 depicts an overview of the system design.

### 3.1. Hardware Description

**Figure 2:**
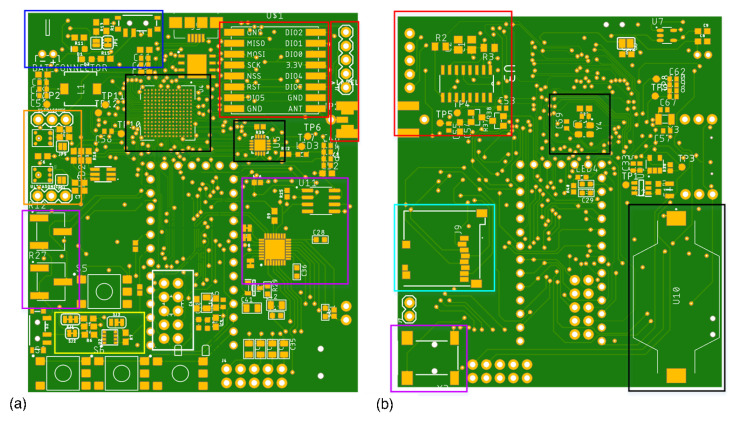
Multipurpose print circuit board sensor and the highlight modules. Red: LoRa module; Black: bootloader, microcontroller core and real-time clock (RTC); Blue: battery module; Orange: Microphone slots and modules; Cyan: microSD module; Yellow: environmental data module; Purple: audio module and controllers; White: Optical slots. (**a**) Top layer. (**b**) Bottom layer.

The multipurpose sensor is a practical and non-invasive approach for surveying ecosystems, for instance, mosquito habitats, dolphins/whales recordings, songbirds to identify acoustics signatures, etc. The device consists of a single credit-card sized (70 × 61 × 10 mm) printed circuit board (PCB), which contains a side-mounted switch to power the PCB and charge the battery. Other features are the universal serial bus (USB) port to program the board; red, green, and blue (RGB) light emitting diode (LED) for notifications; microSD card slot for the data logger; jack for the recording sound or real-time listening; real-time clock (RTC) and LoRa antenna for communications purposes. The device captures sound through two drill holes located at each silkscreened MEMS microphone symbol on the top PCB layer; see Figure 2a. For the optical system, we have eight general-purpose I/O GPIO pins on the top layer for straightforward access. These pins create the option to plug external customized optical systems that interface with the board, allowing users to add hardware modules that expand the board functionality. In addition, the restore button present in the prototype enables the possibility of entering into programming mode, which lets the users upload and update the board code by USB through the Teensy Loader plugin. The programming software can be the Arduino Integrated Development Environment (IDE) (www.arduino.cc, accessed on 20 July 2022) or other platforms with C/C++ programming language (e.g., Microsoft Visual Studio (www.visualmicro.com, accessed on 20 July 2022)).

A multiplatform development environment with multiple advanced features such as PlatformIO IDE (www.platformio.org/platformio-ide, accessed on 20 July 2022) is a helpful tool to program this prototype and recently the CircuitPython (www.circuitpython.org, accessed on 20 July 2022). In addition, the Teensy Loader plugin allows the user to upload code in HEX file format into the flash memory.

The device supports onboard adjustment of volume and gains control, and the sampling rate can go up to 44.1 kHz. A standard feature used in this IoT sensor board is that the audio can be played locally in the field to check the quality, and species can be identified by biologists and experts. The prototype recorded the acoustic sound as 16-bit uncompressed waveform audio (WAV) files into the MicroSD card as a backup.

Figure 2 depicts an overview of our prototype’s most important modules (highlight).

The eight most important modules are composed of the following components and features:

- LoRa Module (Red):
  - RFM95 transceivers
  - Antenna connector
  - Debug pins
- Acoustic Module (Orange):
  - Two ultrasonic microphones on board
  - Two extra slots for microphones, ultrasonics or hydrophones
- Environmental Module (Yellow):
  - BME280 sensor
- Optical module (white):
  - GPIO slots
- Audio module (purple):
  - SGTL5000 chip;
  - Jack connector;
  - Volume control between 0 to 1.0, volume more than 0.8 is too loud;
  - Mic gain control between 0 to 62 dB;
  - W25Q128JV Memory chip;
- Memory module (Cyan):
  - microSD slot;
- Core module (black):
  - MKL02Z32VFG4 Bootloader chip for DIY and easy programming;
  - ARM Cortex-M7;
  - Real-time clock (RTC);
  - Random Acess Memory (RAM);
- Battery module (blue):
  - Battery connector;
  - USB type B mini connector;
  - Battery notification status (LED);
  - 300 mA, 500 mA or 800 mA charger current (user definition);

### 3.2. Power Management

Any 4.2V–6.5V DC supply can power the device (5 V USB recommended) since an internal voltage regulator converts the DC supply to a regular 3.3 V. An internal N and P-channel MOSFET array will disconnect the DC supply when a USB power is connected to reduce the load. In addition, when the device operates in high capacity or high outside temperature circumstances, an inside block controls the charge current automatically to safeguard the device.

When a power supply (e.g., USB or DC supply) is connected and the battery voltage is below 70%, the device enters the trickle charge mode, not given enough current to run the device. On the other hand, if the battery voltage is more elevated than 70%, the charger goes to the bulk charge mode. For example, when it reaches 4.2 V, the charger goes to the constant voltage mode until the battery is full or when the current drops 1% of the programed value.

The battery charger is fixed at 4.2 V with 1% accuracy, a green LED indicates charging, and a yellow LED means power is supplied to VIN (chosen by the slide switch). In addition, it would be better to check the USB’s good power first, which is helpful for alerting users about noise on the USB line, a frayed or damaged USB cord, or the wrong USB input voltage. The device has an inner soft-start circuit that minimizes the maximal instantaneous current. The charge current varies from 0 to the full scale in 100 µs.

The programed charger provides the option of 300 (label JP3), 500 (default-label JP1), or 800 mA (both labels) charge current. This current selection needs to be made, considering the maximum load the lithium battery can handle. Therefore, choosing a charging current below the battery’s capacity is mandatory. For example, if we have a 3.7 V lithium battery with 400 mAh, the current selected in this case would be 300 mA and not more than 400 mA.

**Figure 3:**
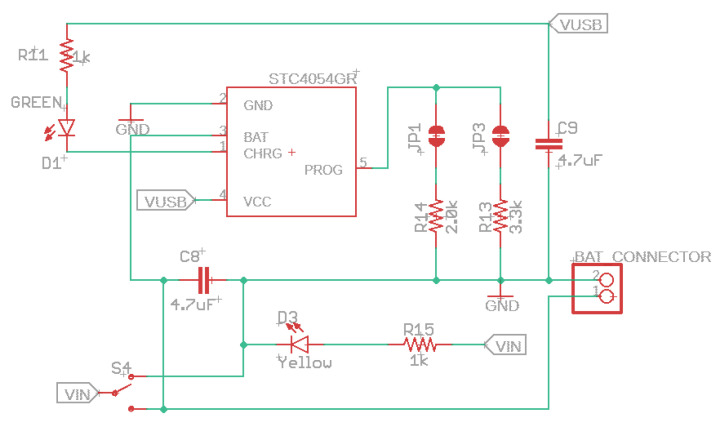
Built-in linear battery charger current up to 800 mA schematic.

For 300 mA, close the solder jumper labeled JP3 and cut the trace between the solder jumper pads marked JP1. For 500 mA, solder the JP1 and cut the trace JP3. For 800 mA, close both soldering jumpers. See Figure 3.

### 3.3. Audio System

**Figure 4:**
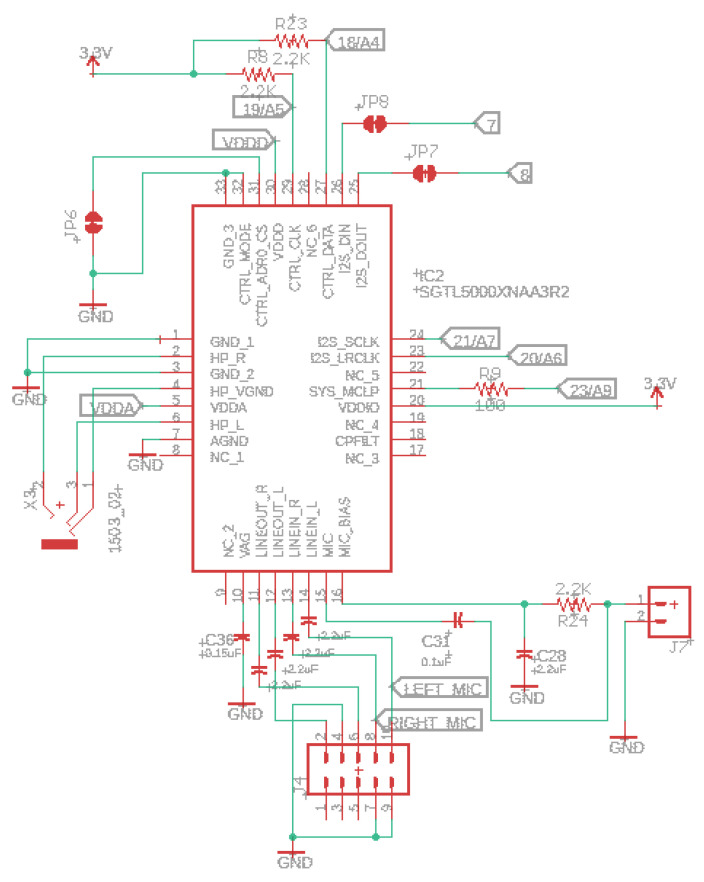
Audio schematic through SGTL5000XNAA3R2/SGTL5000XNBA3R2.

This subsection explains the audio system implemented behind our IoT device solution through the SGTL5000 chip, one of the smaller components in the market. This unit is a low-power stereo codec with a headphone amplifier from NXP. It provides a complete acoustic solution for developments needing stereo lines I/O, mono microphone IN, and digital I/O GPIO; see Figure 4. NXP derives its architecture from best-in-class. As a result, the SGTL5000 can achieve very high performance and functionality with ultra-low power.

This audio chip connects to the processor ARM Cortex-M7 using seven signals. To control the chip and modify parameters, we have the I2C pins (SDA and SCL). The I2S pins for audio data (TX and RX), and finally, three clocks, LRCLK (44.1 kHz), BCLK (1.41 MHz), and MCLK (11.29 MHz). In our device, the SGTL5000 audio chip works in “slave mode”, where all its clock pins are inputs.

Figure 4 shows the audio schematic supporting stereo headphone, stereo line-level input and output, and mono microphone input. Since SGTL5000XNAA3R2 is scheduled for obsolescence and will be discontinued by the manufacturer, the alternative will be SGTL5000XNBA3R2 or SGTL5000XNLA3R2, a perfect compatible successor with no need to change the PCB layout.

One advantage of this chip is the adjustable power architecture at the lowest cost, letting the system minimize power consumption and maximize performance. For example, to have the maximum power in the headphone output level, the VDDA pin runs at the lower voltage possible. On the other hand, for the highest performance, the VDDA pin should run at 3.3 V. Therefore, this lower voltage is used for most applications to achieve the best performance and power consumption combination.

**Figure 5:**
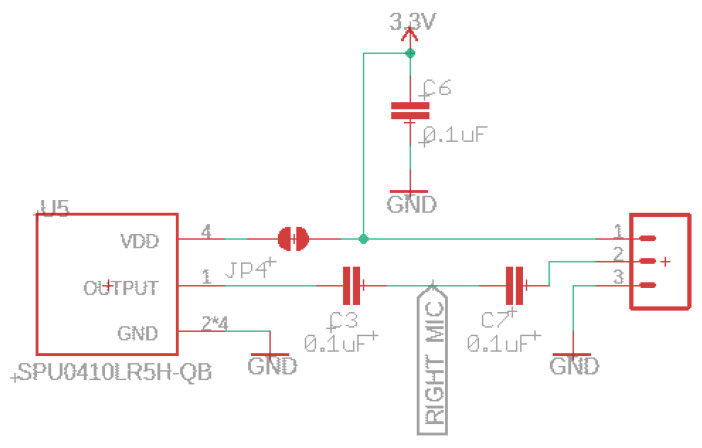
Built-in ultrasonic microphone schematic.

The SGTL5000 audio chip allows you efficiently add high-quality 16-bit sampling rates between 8 and 44.1 kHz. The audio circuit starts at the microphone input signals and is routed straight to the audio board analog peripherals (label RIGHT_MIC); see Figure 5. The SPU0410LR5H-QB is a miniature and low-power microphone with high performance. This acoustic chip has a low noise input buffer, an output amplifier, and a usable ultrasonic response up to 80 kHz. On board this prototype, we have the analog and digital gain/volume, allowing easy setting adjustments. Other microphones can be connected via the 3-pin header and unsolder the JP4 jumper.

The compatibility Teensy Audio Library (www.pjrc.com/teensy/gui/index.html, accessed on 24 July 2022) allows you to forward the microphone input to the output headphone directly with high quality or through the line-out pins, using it simultaneously with other functions. In addition, the toolkit of audio design objects enables the creation of all types of sophisticated acoustic signals and other applications easily. For example, play multiple sound files with some audio effects, mix various streams, and create synthesized waveforms.

### 3.4. Memory

**Figure 6:**
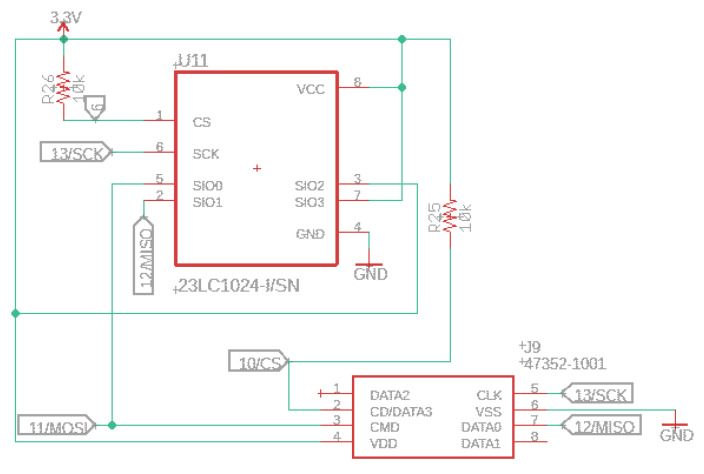
Audio RAM chip memory schematic.

The 23LC1024 RAM chip is added on the top layer side and integrated with the audio circuit module. An essential feature of this unit is the possibility of playing audio files through the SerialFlash library. This chip has considerably lower access latency than standard SD cards, allowing multiple sounds to be recreated simultaneously. A built-in SD socket will enable the user to increase the data storage; see Figure 6.

This component, combined with an IMXRT1062 processor and Winbond flash memory, permits us to make a robust and programmable multipurpose sensor that is fully compatible with microcontrollers such as Teensy 4.0 or Teensy 4.1.

The prototype has a 2Mbyte of flash memory planned for holding code, arrays, and read-only variables. In addition, we may use a sliced piece of the memory for file storage through the recommended LittleFS library. In our case, the first 64K is reserved, where 60 k is for EEPROM emulation data and 4 k for the LED blink restore program. The Random Access Memory has a total size of 1024 K, and its slide is in two parts, generally used for variables and data. The first slide is a tightly coupled memory accessed for higher performance (e.g., accessing the standard variables). The second part is optimized for access, where extensive arrays and data buffers are typically set, making this IoT device optimized for audio with 32-channels.

**Figure 7:**
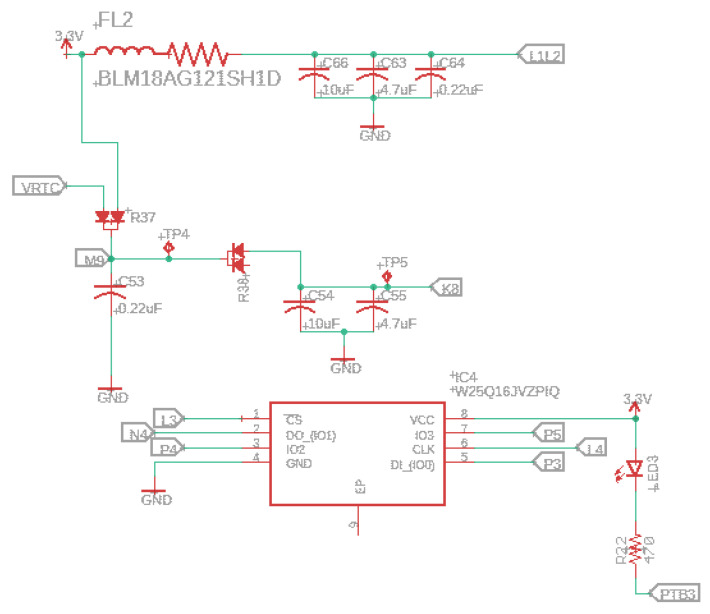
Serial flash memory schematic.

The W25Q16JV (16M-bit) serial flash memory delivers a storage solution for designs with limited space and power problems (e.g., conservation issues). This memory series presents flexibility and performance exceeding the standard serial flash units. The 25Q series is ideal for copying code from nonvolatile to RAM and running code directly from Dual/Quad SPI. Another essential feature is the storing of audio and information data. The unit operates on a single 3.3 V supply with an operating current of 4 mA and 1 µA for power-down consumption. Figure 7 presents the serial flash memory schematic.

### 3.5. Optical System

**Figure 8:**
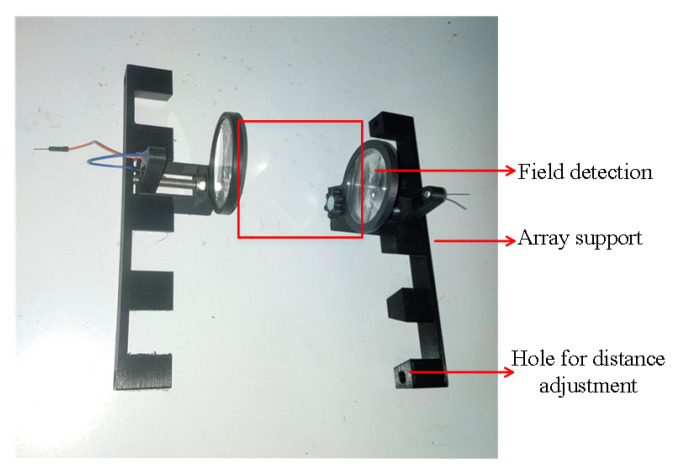
Experimental arrangement from 1 to 4 Fresnel lens.

This subsection describes the optical system design and characterization to use with our prototype for detection purposes (e.g., mosquitoes or other small insects). We detect the insect by identifying the fly’s transiently reduced shadow when passing through emitted light. Our setup is shown in Figure 8.

**Figure 9:**
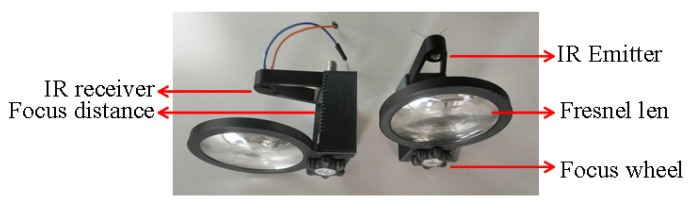
Optical sensor infrastructure.

We placed the infrared emitters (e.g., L-53F3C at 940 nm) and photodiodes (e.g., BP104FS-Z at 940 nm) in the array support, capable of incorporating a group of four emitter–receiver systems. In addition, the Fresnel lens group has a focus wheel so that the IR units can be centered with the Fresnel lens, as shown in Figure 9. The LEDs emit infrared light through the emitter Fresnel lens group with a diameter of 50mm. A set of IR lights together forms a parallel laser field (field detection area). Fresnel lenses are responsible for directing the lasers to the receivers. All lenses have a focal length of 40 mm.

**Figure 10:**
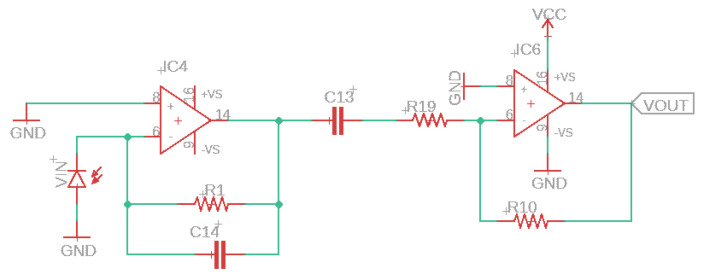
Current to voltage converter (first OPAMP) and photodiode amplifier (second OPAMP) circuit.

The optical system works while the insect passes through the field detection, called field of view (FOV); the electric current of the photodiode (VIN) varies proportionately due to the shading of the light through a transimpedance amplifier. Figure 10 depicts the circuit and how the photocurrent variation is amplified. The photodiode acquires a higher electric current signal when no insect passes via the field detection, meaning that the photocurrent passing through the first amplifier (AC-coupled) is zero. In the opposite situation, when a flying insect passes via the field detection area, the strength of the infrared light obtained by the receiver is modulated, ensuing in an oscillating electric current amplified by the second OPAMP (IC6) presented in Figure 10.

**Figure 11:**
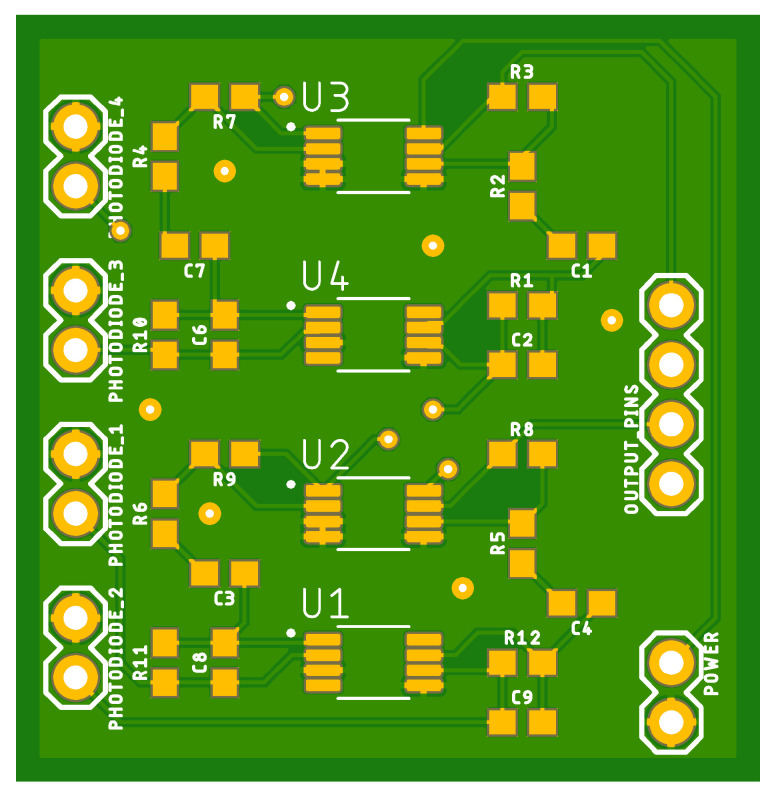
Optical board.

Figure 11 depicts the shield responsible for connecting all four photodiodes to our multipurpose device.

**Figure 12:**
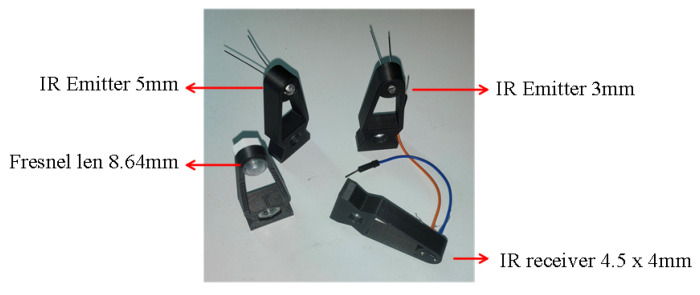
Different types of support.

To create a more sophisticated architecture for insect optical field detection, we build four types of adaptors (see Figure 12 for the optical system present in Figure 8); two supports for the infrared LEDs of 3 and 5mm, one for the photodiode and another for a small Fresnel lens (e.g., CMS442CTP), see Table 1. Hexagon head screws are recommended to connect all the components.

### 3.6. LoRa Connectivity

**Figure 13:**
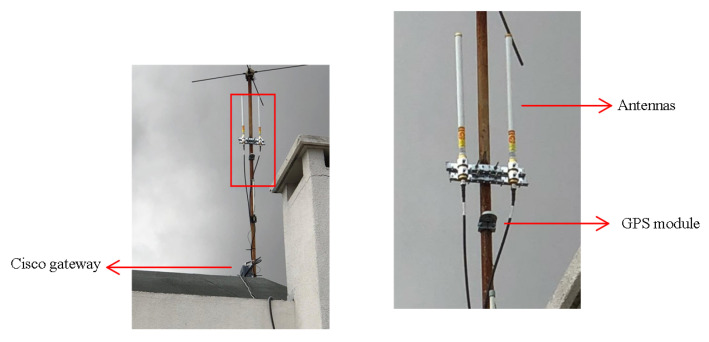
LoRa communication gateway infrastructure.

The multipurpose device plans to use LoRa as a communication technology. The unique capabilities of LoRa are ultra-low-power, high performance, and affordable long-range connectivity covering entire areas or cities with just a few base stations connected to the Internet, no longer requiring the implementation and maintenance of devices as in traditional mesh networking such as WiFi. These factors make this technology chosen, as it fulfills the requirements for implementation in remote places (e.g., distance, maintenance, and energy consumption), making it one of the defacto standards in IoT approaches. Regardless, our IoT device occasionally connects to the closest base station to send short pieces of data collected by the base station and sent to the user-defined server. However, sending audio files becomes unsustainable because this technology is a low-bandwidth solution. Consequently, the classification needs to be conducted on the device itself. In addition, the user needs to consider the environmental conditions since LoRa has some performance loss when there are great obstacles to the transmission and reception of the RF signal (e.g., mountains, forests, and large buildings). To incorporate these environmental factors and the impacts into LoRa’s connectivity, it is recommended to use the Radio Mobile Software (http://radiomobile.pe1mew.nl/, accessed on 24 July 2022), namely for forest areas. This step will ensure good communication and implementation of the final system instead of hand-picking optimal deployment positions. Figure 13 shows the Cisco Wireless Gateway infrastructure for LoRaWAN used to test this prototype.

The RFM95W transceivers feature used in our sensors can reach a sensitivity of around −148 dBm employing a low-cost crystal and a set of cheaper materials. This transceiver with high sensitivity fused with an integrated power amplifier (+20 dBm) makes it an optimal price–cost relation, ideal for any application needing long-range or robustness connectivity.

## 4. PCB Components

The list of materials for the PCB construction is composed of the general hardware with acoustic sensors and sliced into two versions of custom functionality: the optical and hydrophone-operated versions. Table 2 contains unit pricing to buy enough components to assemble a single device, a portion of 100 or 1000 with acoustic sensors incorporated. Ordering pieces and PCBs in large quantities results in a more considerable cost-saving per prototype, which is essential for large-scale deployments.

Table 3 includes the bill of materials to purchase components sufficient to build a kit unit (array of 4 optical sensors).

Table 4 includes the kit unit materials to build yourself one hydrophone sensor for aquatic activities and attach it to our IoT device. This sensor can be soldered to the J7 connection, as shown in Figure 4. To isolate the piezo, we can use epoxy for plastics, which is excellent for waterproofing and ensuring a solid construction.

## 5. Validation and Setup

This section describes how to validate the proper build of the multipurpose sensor hardware and its performance. The board will work if the IMXRT microcontroller and a flash memory unit are paired correctly with the MKL02 bootloader. Therefore, the hardware is created with a microcontroller, so the validation involves inspecting the functionality of the peripherals, which includes the USB bootloader, SPI interface that communicates with the microSD card, I2S communication between audio circuit and microprocessor, and the correct reading from the microphones, hydrophone, and optical system.

### 5.1. Bootloader Validation

The microcontroller chip has special power-up sequence requirements to work correctly. The validation of the boot loader is accomplished with the following steps:

1. The multipurpose microcontroller has a boot loader (MKL02Z32) pre-programed at the factory.
2. When plugged in via USB, power arrives at the USB voltage regulator built in the microcontroller unit and the XC6210B332MR regulator through VIN or VUSB.
3. The USB voltage controller turns on, and VDD_USB_CAP charges up to 2.5 V. At this moment, the supply voltage input (Secure Non-Volatile Storage and Real-Time Clock (SNVS_IN)) regulator receives power.
4. The SNVS regulator turns on and creates 1.1 V at VDD_SNVS_CAP.
5. The IMXRT power management module turns on the Phase Locked Loop (PLL) and analog regulators (1.1 V at NVCC_PLL and 2.5 V at VDD_HIGH_CAP).
6. Once power and the regulator are stable, the PMIC_ON_REQ pin has 1.1V to request main power.
7. The XC6210B332MR regulator turns on and supplies the entire main board with 3.3 V.
8. After 3.3 V is stable, the boot loader chip is turned on and drives DCDC_PSWITCH to high.
9. The IMXRT inside the DCDC buck converter starts producing 1.15 V power for the CPU and most internal circuits. This voltage can be automatically adjusted as needed for higher speeds (>528 MHz).
10. A PC will recognize a fully assembled device at this stage.
11. Start the flashing by USB using software (Section 3.1) for this test.

**Figure 14:**
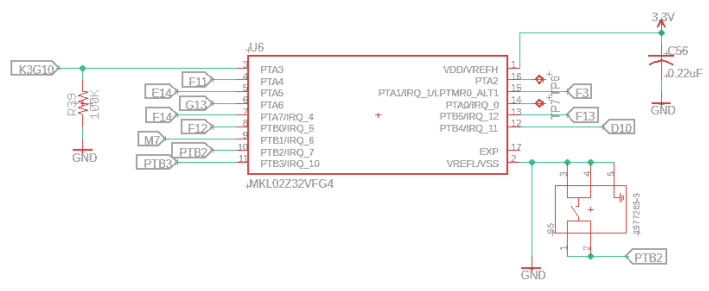
Bootloader schematic.

An important note is that the board will run a LED blink program by default when the first time the restore button is pressed (PTB2) with a size of 4 k. After pressing this button for 15 s, the flash memory is erased and will copy the default blink program to the first part of the flash chip. This restore procedure will only work if the user has already pressed the button at least once for the regular bootloader/program. Figure 14 depicts the characteristics of the bootloader and restore button.

This bootloader has released a new feature and can be used in our prototype, called secure mode (www.pjrc.com/teensy/td_code_security.html, accessed on 25 July 2022) state. This feature’s main benefits are that the program code kept in the flash memory is encrypted, the code will run if your key is used, and JTAG access is disabled.

### 5.2. Peripheral Validation

We validate the peripherals using the most straightforward programs to make a WAV file sound recording to a microSD card or forward the sound directly to the headphone jack. This procedure is essential to identify hardware issues quickly. For example, skips in the recorded sound indicate a slow/poorly connected SD card or incorrect pitch indicates problems with sample rate. Other peripheral tests can be archived using the programed files in Table 5. You can upload the .hex files through the Teensy Loader plugin for testing purposes. For the optical connections, you need to read the GPIOs and check if the infrared field changes when passing through them.

**Figure 15:**
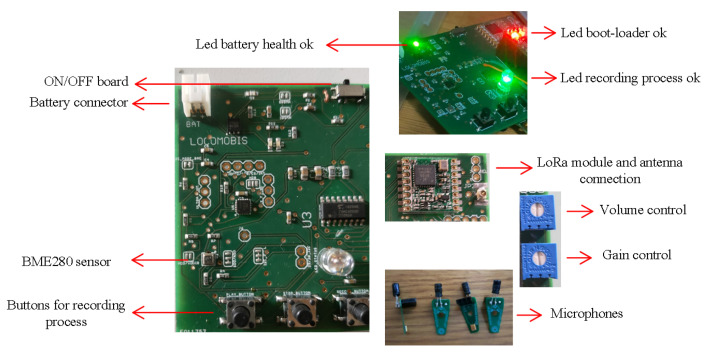
Prototype and principal test components.

For recording validation tests, it is helpful to download the free open-source, cross-platform audio software, Audacity (www.audacityteam.org/download, (accessed on 24 July 2022)), to check the audio files. Figure 15 shows an overview of the prototype and its components. Validation of the peripherals is achieved through the following subsections.

### Hardware Test

**Figure 16:**
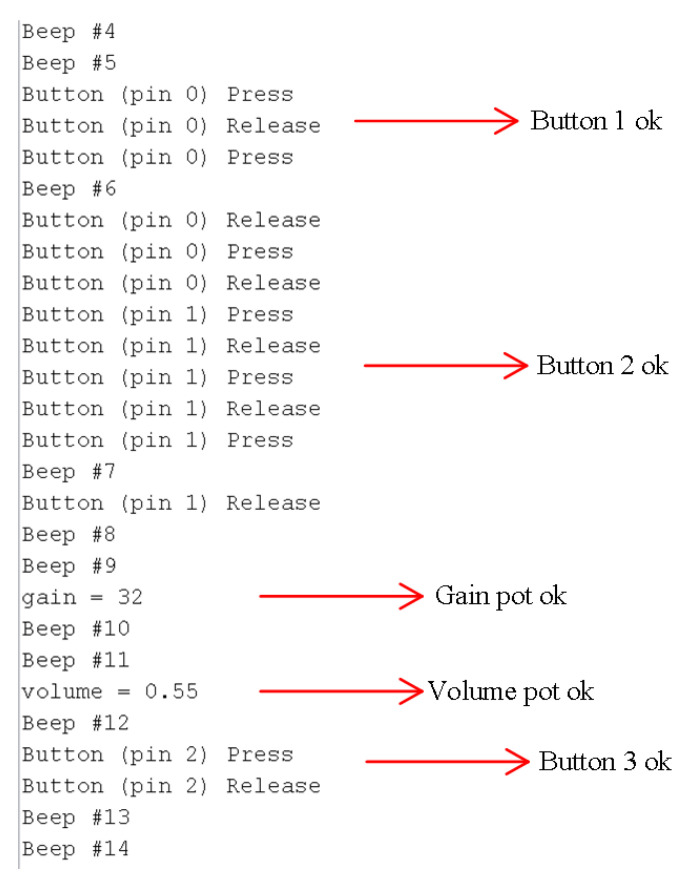
Mechanical peripherals test.

To test the functionalities and the peripheral of the device, a simple beeping program can be pre-loaded on the Teensy, and this will create sound and print the information to the serial monitor when plugged into a PC with the appropriate software. The file created a sinusoidal signal with 500 Hz and 0.9 amplitude. Plugin the headphone in the jack slot, and we should see a beep message for each headphone beep. The last step is pressing the buttons (e.g., play, record, and stop) and turning the two potentiometers on the PCB prototype. See the hardware response in Figure 16. The functionality of the buttons is to play, record, and stop recording/playing the audio, in this case.

**Figure 17:**
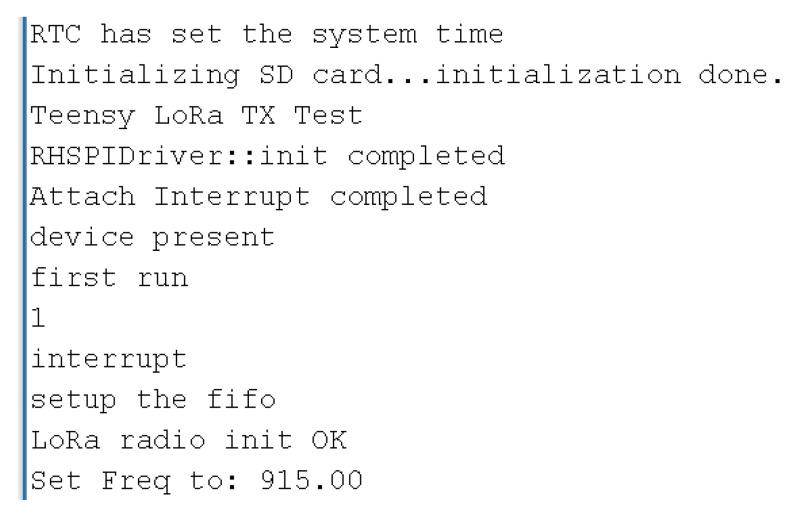
LoRa, RTC and SDcard test.

Figure 17 shows the validation print for the connections: LoRa module, RTC and SDcard working properly, using the file “LoRa_Test.ino.hex” present in Table 5.

**Figure 18:**
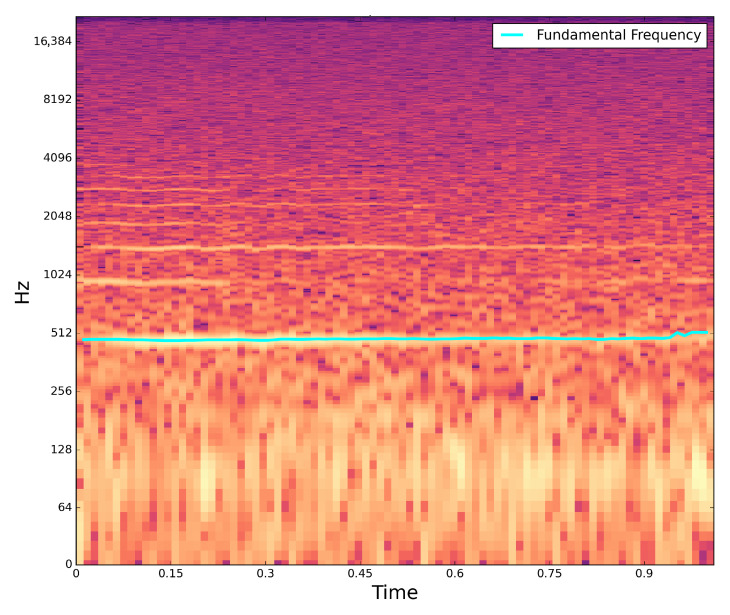
Audio file from an *Ae.Aegypti* mosquito with a mean frequency of 481 Hz.

Validation of the acoustic peripheral can be achieved by pre-loading the corresponding file. Figure 18 shows a spectrogram for the audio file recorded to the SDcard slot with a sample rate of 44.1 kHz.

The same acoustic process can be made for the optical module, checking if the infrared field changes after causing a disturbance.

### 5.3. Energy Management

Low power consumption is critical for long-term monitoring applications, especially in remote areas. The average consumption of the sensor depends on the number of detections, and the battery capacity. The user requirements will dictate the lifetime and the need for battery replacement.

The user can set the optical module, acoustic recorder, sample rates, gain, volume, environmental readings, time schedules, LED functionality, battery charger, and LoRa communication. We recommend that a lithium-ion polymer battery of 3.7 V power our prototype. Battery life can be optimized through efficient consumption management strategies. All the parameters can be adjusted for specific target activities.

Table 6 presents the energy measuring of a 3.7 V battery in function on the prototype functionality.

Table 7 depicts examples of the configuration guidelines for different applications and the corresponding battery lifetime for a lithium battery with 6 Ah. For the mosquito approach, we have a 100% active time because the optical system always waits to trigger the audio processing stage. The approach with LoRa communication has a higher consumption than the Dolphins and Soundscapes applications. The greater the number of detections and the active time, the greater the consumption, consequently reducing the battery life.

## 6. Preliminary Tests

This section presents the validation and characterization of flying mosquitoes through acoustic sensing using audio features and machine/deep learning approaches. The optical system will use a wing beat frequency technique, which is an efficient solution to differentiate these fly insects. We will discuss an example of the mosquito species.

All the preliminary experiments were conducted in a controlled laboratory environment to demonstrate the feasibility of insect detection using the combined audio-optical principle. First, the optical procedure focuses on the detection to trigger the recording, and then the acoustic stage classifies this type of species understudy.

Here, we summarize the preliminary laboratory experiments to test the multipurpose prototype.

- Data collection:
  - Recording setting: 32 float format, 1 channel (mono), 8 and 16 kHz sampling rate, WAV file format
  - Microphones: ultrasonic Mic SPU0410LR5H-QB
  - Container: chambers shaped container wrapped around with a net, size: 25 × 25 × 25 cm
  - Temperature: 23–25 ${}^{\circ }$C
- Pre-processing:
  - Separate the portions of the recordings that contain only the segment of mosquito flight tones
  - Frames: windowing: 0.3 s; overlapping: 0.15 s
  - Spectrogram Transformation: Short-time Fourier transform (STFT)
  - Determine the fundamental frequency with probabilistic YIN algorithm (Frequency ranges)
  - Extract Features: 34 signal features

### 6.1. Optical Experiment

After detecting the event within the frequency range of the mosquitoes, where males (664–956 Hz) have higher frequency ranges than females (480–620 Hz), the pYin algorithm (pitch detection method) [26] is applied to have a degree of confidence about the frequency detected through the harmonic rate parameter.

**Figure 19:**
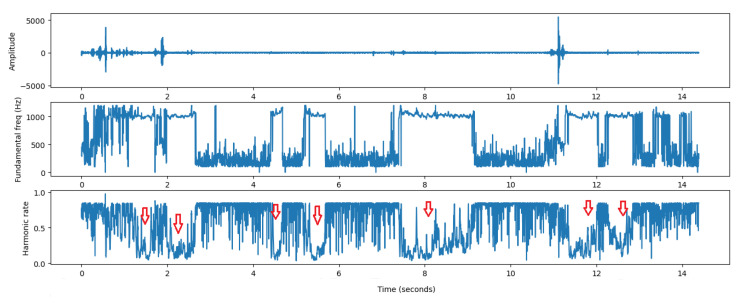
Fundamental frequency estimation and harmonic rate value using pYin algorithm for mosquito counting (red arrow).

The lower this value, the more likely that the sound corresponds to a mosquito; see Figure 19. This method shows how the frequency is distributed along the spectrum. Its input is the vector of the event (size and time of the data) and the output is the pitch range within the detected time interval, as well as the harmonic rate of the frequency in the event frame. This algorithm is relatively robust in terms of performance and can be used in embedded real-time devices.

In the optical recording file shown in Figure 19, we can count at least four *Aedes Aegypti* mosquitoes. This rudimentary approach to counting mosquitoes at each event, where one event (red arrow) or very close consecutive events with the same frequency variation is equal to the presence of one mosquito.

The algorithm provides a distinct method of identifying the presence of mosquitos in the laboratory and urban environments while largely neglecting other sounds. The optical system currently records the shadow, and as a future prospect, the user may consider the backscattered light, which is richer in harmonics and could be more practical for insects application because it emits light in the open space that is backscattered by the wingbeat and does not require the insect to pass through the emitter–receiver pair.

### 6.2. Acoustic Experiments

For the user to replicate our experiment, we used the dataset present in [27]. We extracted the audio features from the audio files used in that work and applied the various machine learning techniques for the species *Aedes Aegypti* and *Culex Quinquefasciatus*. This process allowed us to generate signatures for each mosquito species and match them to the audio segments.

We extracted 34 signal features: zero crossing rate, energy, entropy of energy, spectral centroid, spectral spread, spectral entropy, spectral flux, spectral roll-off, 13 Mel-Frequency Cepstral Coefficients (MFCCs), 12 chroma vectors, and chroma deviation.

Then, we applied six machine learning methods: k-nearest neighbor (k-NN), Support Vector Machine (SVM), SVM-RBF, random forest, gradient boosting, and extra trees. Table 8 shows the evaluation for the six machine learning models and the value of the input parameter that optimizes the accuracy performance measure. The inputs to these models were the thirty-four signal features of the audio signals at 8kHz. The classifiers were evaluated with stratified 20-fold cross-validation. As shown in Table 8, the Grading boosting has the highest accuracy and F1-Score, followed closely by the extra trees and SVM with linear kernel. We used the library present in [28] to perform the classification system.

The training group was composed of 80% of the number of samples, 10% for testing, and 10% for validation of each species: Female *Aedes Aegypti* and Female *Culex Quinquefasciatus*. To implement this approach in the prototype, we can use the Micropython platform (www.micropython.org/, accessed on 24 July 2022) compatible with our device through the generated files of each model to classify these two species; see Table 9. Furthermore, we recommend the SVM with a linear kernel because it is the most lightweight model compared to the size of the other machine learning models and fits in the microcontroller memory.

Following the example, in [29,30], we also implemented a Convolution Neural Network (CNN) to test the prototype’s robustness in terms of deep learning methods using the TensorFlow lite library.

**Figure 20:**
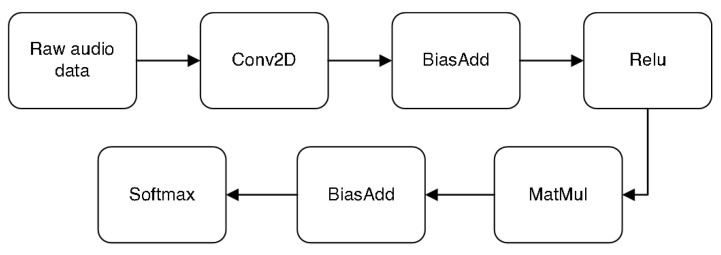
Convolutional model aimed at microcontrollers.

The total number of steps to training was 4000 with different learning rates. Therefore, the first 3000 with 0.001 and the last 1000 with 0.0001 learning rate. The model architecture chosen was the ‘tiny_conv’ option and the spectrogram processing mode ‘micro’. As shown in Figure 20, the network starts with a 2D convolution layer that takes the raw audio data in Tensor shape. In the output of the first block, we apply Bias and then pass through the rectified linear activation function (RELU). During training, a dropout node is introduced after the RELU, controlled by a placeholder. The output of the activation layer passes through the multi-layer dense block. Finally, the softmax prediction process converts a vector of four real numbers into a probability distribution of four possible outcomes. The model has as four output labels (unknown, silence, *Aedes* and *Culex*).

Table 9 also shows the float and quantized models used in the prototype with a size of 68,048 and 18,712 bytes, respectively. To help train and test this model, we added several 16 kHz WAV files of various types of background noise. Both models have a similar accuracy of 88%.

Table 10 summarizes the classification algorithm implemented in the device to classify 81 recorded audio files where we have 11 false positives and 70 true positives. The input data are contained in an audio file with a 16 kHz sampling rate and a 1-second time frame after being activated by the optical system.

This does not produce particularly accurate results in a noisy environment, but it is designed to be used as the first stage of a pipeline, running on a low-energy piece of hardware. We can train a standard convolution model to produce fairly good quality results, involving many weight parameters and computations for more accurate results.

Without environmental noise, we can improve the classification by having an accuracy of about 90 % for *Aedes Aegypti* and *Culex Quinquefasciatus* using a 300 ms window instead of a 1s time frame. One way to reduce this false positive is by combining noise-canceling microphone procedures and using well-known audio features in speech recognition.

The optical-acoustic combined methods are a way of double verifying the insect species. The first one verifies the frequency ranges of the detected species, “telling” the acoustic which kind of species to expect. Since some species have similar frequencies, the acoustic will dissipate those doubts. One advantage of this combination is that the optical process is a low-power system that allows the prototype to live longer, just triggering the acoustic when necessary.

## 7. Typical Applications

The creation of our multipurpose device has been driven by international demand from the environmental monitoring and conservation communities. Since deploying the first version of the sensor [31], we have developed numerous partnerships worldwide, testing the device for diverse applications, especially targeting mosquito monitoring. One collaboration with the University of Mahidol in Thailand, Spatial Cognition Center (BSCC), in partnership with the University of Bremen, combined our sensor with street view images to detect potential breeding containers. In this research, the sensor was deployed in the Rajanagarindra Tropical Disease International Centre (RTIC) [31] to validate the risk mapping from seasonal-spatial models in which the target variable dengue incidence was explained using weather and container variable predictors. Another collaboration with the University College London further explores the weather variables by incorporating additional sensors (i.e., physicochemical parameters) that correlate mosquito counting with water quality parameters that impact the presence and abundance of mosquitoes. This approach aims to improve the efficiency of real-time mosquito abundance modeling and predict high-risk areas of infestation and breeding. During 2019 and 2020, 10 prototype devices were deployed in Madeira Island with the default recording program [27]. A new deployment is planned in Madeira Island and also in Brasil.

However, our device is not restricted to mosquito monitoring. Ecoacoustic monitoring is growing with multiple applications in different domains, including the monitoring of protected and invasive species in the wildlife (e.g., bats, birds, bees, toads, etc.) [32,33,34,35], soundscape analysis [36,37], biodiversity conversation [38], environmental surveillance [39,40] and ocean monitoring [41,42]. In addition, the optical monitoring feature enhances the potential of identifying insects [12] since it is immune to ambient noise, unlike acoustic approaches. Despite acoustic noise cancellation techniques, we can change the necessities for hardware configuration and program as we like for each application. The most critical sound characteristic to capture is the range of audio frequencies (e.g., forming a fingerprint) by the target species. For example, manual surveillance techniques are time-consuming, such as trapping, and manual identification is labor, time, and cost-intensive [43,44]. Furthermore, to register the source signal, the sampling rate must be at least twice the frequency created by the organisms, called Nyquist frequency. The audio will not record any sound above this criteria.

The speed of the SDcard is essential for multiple access (e.g., playing various files simultaneously) and running machine learning algorithms. Therefore, we recommend an SDcard with low latency for non-sequential access and a memory capacity of 32 GB. As well as programmable devices, other parameters can be adjusted for a particular type of application. For example, the duty cycle routine, sleeping and timed recording schedules through the RTC sensor can be adjusted for specific target species or activities (e.g., bat echolocation activity), which will save power and memory required for each deployment. All indication LEDs can be turned off to prevent unwanted attention (e.g., night deployments). Gain and volume settings can be changed to adjust for different background noise levels during recordings. The possibility of being able to record and listen simultaneously with headphones allows this adjustment to be more efficient when the environment noise is unknown to prevent audio distorting.

Very short sounds can be stored directly into the program memory. Analyzing sounds from the microcontroller memory has a significant advantage: it is much quicker and more energy-efficient, allowing dozens of sounds to play simultaneously without reaching microcontroller resource limits. Then save the data on the SDcard as a backup and send the information over LoRa protocol. This avoids the storage of useless files, saving memory space.

Typically, bioacoustics analysis has measured features such as waveforms, spectrograms (e.g., signal frequencies), power spectra, and selected measurements such as duration and fundamental frequency. However, nowadays, several new software tools allow for automated parameter extraction. Machine-based feature extraction algorithms can provide a new world of signal feature sets [45,46]. Some examples include spectral features and cepstral coefficients commonly used in human speech processing and recognition [47].

Nowadays, we have the TensorFlow Lite (www.tensorflow.org/lite/microcontrollers, accessed on 25 July 2022) for microcontrollers, and it is designed to run machine learning models on these microdevices with only a few kilobytes of memory. The minimum requirement to run multiple basic machine models is 16 KB on an Arm Cortex M3. It does not require operating system support, standard C or C++ libraries, or dynamic memory allocation to run this feature. A solution is compatible with our prototype, which has digital signal processor (DSP) instructions providing plenty of computational power for real-time applications, unlocking the chance to undertake advanced audio-reactive projects.

## 8. Conclusions

This paper describes the hardware design of a multipurpose, low-cost, IoT-based device for biodiversity monitoring that combines acoustic and optical sensors. We have provided the location of the optical 3D print support design files, code test files, and instructions on deploying them. After hardware assembly, we described the procedure for the bootloader and peripheral validation processes. In addition, we provide the guidelines to test the hardware functionalities and how to deploy our IoT working device. The scientific community can use this device for different biodiversity monitoring approaches and conservation tasks, such as studying wildlife vocalization behaviors. Still, it can also be applied to detecting bat signals, insect applications (e.g., mosquitoes and pollinators), and ocean monitoring purposes (e.g., dolphins and whales).

Furthermore, the cheaper construction components make this device a low-cost product, and the small size makes it easy to transport and implement in challenging places. In addition, high computational operation and simple programmable devices also increase deployments’ scalability in rural and forest areas using LoRa technology through our battery-powered device, offering to the industry a diverse set of applications. The battery and memory consumption predictions can be recorded to estimate battery life, allowing planning and scheduling to replace batteries since the data are sent over LoRa protocol, increasing the feasibility of implementing low-power devices for long-term deployment in the field. As shown with ongoing deployment, our device enables more important conservation research questions to be answered with all-in-one sensors technology. Using machine learning on low-cost microcontrollers creates a new age for smart devices. After training, we can deploy machine/deep learning algorithms onto our smaller embedded units.

## Figures and Tables

**Table 1:** Optical modules location.

Module	Description	Location ^1^
Led 3 mm	Support for a 3 mm infrared led	v17_Led-support-body-3 mm.stl
Led 5 mm	Support for a 5 mm infrared led	v17_led-support-body-5 mm.stl
Big Fresnel	Support for a Fresnel lens with 50 mm of diameter	v17_big-fresnel-support-body.stl
Small Fresnel	Support for a Fresnel lens with 8.64 mm of diameter	v17_small-fresnel-support-body.stl
Hand adjust	Spin adjustment for hand	v17_adjust-support-for-hand.stl
Photodiode	Support for a photodiode with 4.5 × 4 mm	v17_photodiode-support-body.stl
Support body	Support interconnection rail and hand adjustment	v17_support-bodies.stl
Array infrastructure	Connection rail from 1–4 support body	ArraySupport_Front.stl
Video	Video demo how to adjust the optical sensor infrastructure	Video Demo.mp4

^1^https://github.com/DinarteVasconcelos/Opto-Acoustic-Modules/, (accessed on 29 June 2022).

**Table 2:** Acoustic and multipurpose device bill of materials.

Designator	Quantity	Manuf. Code	Price €	100 ^1^	1000 ^1^	Source (accessed on 24 July 2022)
C1	1	08056D106KAT2A	0.219	0.099	0.099	mouser.com
C2	1	CC0805KRX7R7BB104	0.143	0.048	0.028	mouser.com
C3-7, C11-12, C31	8	0603YC104KAT2A	0.114	0.039	0.023	mouser.com
C8-9	2	06036D475KAT2A	0.152	0.05	0.041	mouser.com
C23, C28, C52	3	CC0603KRX5R7BB225	0.219	0.077	0.047	mouser.com
C10, C27, C29-30, C39, C43	6	0603YC104KAT2A	0.114	0.039	0.023	mouser.com
C32, C54, C66	3	C0603C106M9PACTU	0.304	0.119	0.073	mouser.com
C33, C45-47, C50, C53, C56, C58-60, C64	11	04026D224KAT2A	0.162	0.044	0.026	mouser.com
C34-35, C37-38, C40-42	7	CC0805KKX7R8BB225	0.352	0.136	0.085	mouser.com
C36	1	C0603C154K5RACTU	0.352	0.123	0.101	mouser.com
C44, C49, C55, C62-63, C68	6	GRM155R60J475ME47D	0.114	0.042	0.028	mouser.com
C48, C51	2	04023C103KAT2A	0.095	0.01	0.006	mouser.com
C57, C67	2	C0402C200J5GACTU	0.409	0.173	0.109	mouser.com
C61, C69	2	C0402C120J5GACTU	0.095	0.017	0.01	mouser.com
CN1	1	S2B-PH-K-S(LF)(SN)	0.0889	0.0889	0.0728	farnell.com
D1	1	HSME-C191	0.599	0.214	0.167	mouser.com
D3	1	ASMT-RF45-AN002	0.466	0.203	0.117	mouser.com
F1	1	MF-PSMF050X-2	0.38	0.233	0.206	mouser.com
FL1	2	BLM18AG121SH1D	0.105	0.04	0.029	mouser.com
IC1	1	AP7313-18SRG-7	0.371	0.187	0.117	mouser.com
IC2	1	SGTL5000XNAA3R2/ SGTL5000XNBA3R2	5.64	4.15	4.02	mouser.com
IC3	1	MIMXRT1062DVL6B	18	12.98	12.97	mouser.com
IC5	1	W25Q16JVZPIQ	0.665	0.591	0.591	mouser.com
J5	1	10104110-0001LF	0.789	0.542	0.451	mouser.com
J9	1	47352-1001	3.94	2.9	2.37	mouser.com
L1	1	CDRH62BNP-4R0NC-B	0.77	0.483	0.328	mouser.com
LED2	1	ASMT-YTD9-0AA02	1.04	0.441	0.348	mouser.com
LED3, LED4	2	ASMT-RJ45-AQ502	0.466	0.203	0.117	mouser.com
Q2	1	DMP2033UVT-7	0.418	0.27	0.163	mouser.com
R2-3	2	CRCW0805100KFKTA	0.152	0.052	0.022	mouser.com
R4-7	4	CRCW06034K70JNEA	0.095	0.014	0.007	mouser.com
R8, R23-24	3	ERJ2GEJ222X	0.095	0.011	0.006	mouser.com
R9	1	ERA3AEB101V	0.333	0.111	0.047	mouser.com
R11, R15-18	5	ERA3AEB102V	0.266	0.095	0.056	mouser.com
R12, R27	2	3361S-1-253GLF	1.59	1.18	0.792	mouser.com
R13	1	ERA3AEB332V	0.333	0.111	0.047	mouser.com
R14	1	ERA3ARB202V	0.77	0.352	0.194	mouser.com
R22, R40	2	CRG0402J470R	0.002	0.0019	0.0018	farnell.com
R25-26	2	CPF0402B10KE1	0.58	0.248	0.136	mouser.com
R28	1	ASC0603-2M2FT5	0.0158	0.0131	0.01	farnell.com
R29	1	BLM18AG601SN1D	0.124	0.039	0.027	mouser.com
R36, R39	2	CPF0402B100KE1	0.205	0.183	0.138	farnell.com
R37-38	2	BAT54C.215	0.171	0.086	0.036	mouser.com
S4, S2	2	MLL1200S	2.4	1.9	1.4	mouser.com
S3, S5, S6, S9,	4	1977263-3	0.208	0.208	0.208	mouser.com
U4	1	RFM95W-868S2	19.57	15.19	13.97	mouser.com
U1, U5	2	SPU0410LR5H-QB	0.789	0.565	0.466	mouser.com
U2	1	BME280	7.21	4.37	3.59	mouser.com
U3	1	74HC4050D.653	0.58	0.37	0.291	mouser.com
U6	1	MKL02Z32VFG4	3.97	2.4	2.03	mouser.com
U7	1	STC4054GR	1.9	1.62	1.62	mouser.com
U9	1	XC6210B332MR	1.01	0.649	0.56	farnell.com
U10	1	SMTU 2032-LF	0.68	0.634	0.521	mouser.com
U11	1	23LC1024-I/SN	2.45	2.4	2.4	mouser.com
X1	1	U.FL-R-SMT(01)	1.28	0.988	0.712	mouser.com
X3	1	1503 02	2.03	1.3	1.16	farnell.com
Y3	1	ABS07L-32.768KHZ-T	1.19	0.96	0.781	mouser.com
Y4	1	TSX-3225 24.0000MF20G-AC0	0.399	0.266	0.213	mouser.com
MainPCB	1	PCB	0.762	0.2563	0.1778	jlcpcb.com
MicroSD	1	Sandisk Ultra-32 GB	5.86	5.29	4.9	bulkmemorycards.com
Total per unit	-	-	109.69	74.50	64.89	-

^1^ Quantity (€).

**Table 3:** Optical sensor bill of materials.

Designator	Quantity	Manuf. Code	Price	100 ^1^	1000 ^1^	Source (accessed on 24 July 2022)
IC4, IC6	4	OPA2380AIDGKT	6.33	4.74	4.51	mouser.com
VIN	4	BPW 34 S-Z	1.11	0.472	0.373	mouser.com
Emitter 3 mm	4	SFH 4350	0.979	0.417	0.33	mouser.com
C13	4	CM03X6S105M10AH	0.266	0.096	0.058	mouser.com
R19	4	CR0603-JW-102ELF	0.095	0.006	0.003	mouser.com
R10	4	ERJ-UP3F7502V	0.228	0.079	0.033	mouser.com
R1	4	ERA-3AEB562V	1.01	0.649	0.398	mouser.com
C14	4	885012006032	0.095	0.027	0.027	mouser.com
Fresnel Lens	8	D50 mm and focal length 40 mm	3.61	1.461	1.461	aliexpress.com
Total supports kit 110 g	1	HATCHBOX 1.75 mm Black PLA 3D Printer Filament	2.479	2.479	2.479	amazon.com
PCB shield	4	PCB	0.76	0.1262	0.0548	jlcpcb.com
Total per unit	-	-	74.85	40.61	37.31	-

^1^ Quantity (€).

**Table 4:** Hydrophone sensor bill of materials.

Designator	Quantity	Manuf. Code	Price	100 ^1^	1000 ^1^	Source (accessed on 24 July 2022)
Piezo sensor	1	MCABT-455-RC	1.94	1.73	1.53	farnell.com
Hydrophone cable	5	Mogami W2549 per feet	0.94	0.94	0.94	redco.com
Total per unit	-	-	6.64	6.43	6.23	-

^1^ Quantity (€).

**Table 5:** Programed test files location.

Module	Description	Location ^1^
Mechanical peripherals	Test the 3 buttons, 2 potentiometers and jack of 3.5 mm	Hardware_Test.ino.hex
LoRa, RTC and SDcard	File to test the communication for LoRa module, RTC and SDcard slot	LoRa_Test.ino.hex
Acoustic peripheral	Record a WAV file and check its parameters	Recorder.ino.hex
Environmental sensor	I2C scan for BME280 communication	BME280Test.ino.hex

^1^https://github.com/DinarteVasconcelos/Opto-Acoustic-Modules/, (accessed on 29 June 2022).

**Table 6:** Power consumption in mA for a lithium-ion battery with 6 Ah.

SDcard Access	Audio Processing	Environmental Readings	Optical System	LoRa Communication	Playing Sounds and Headphones (Volume = 0.5)	Sleep Mode
61	81	54.3	23	110	75.4	7.5

**Table 7:** Simulation of power durability application.

Function	SD Speed	Active Time per Hour	Sleep Time per Hour	Number of Detections	Gain	Average Consumption (mA)	LoRa	Optical	Battery Life in Hours
Mosquito	Class 10	100%	0%	20	Med	73.4	Yes	Yes	65
Dolphins	Class 10	17%	83%	15	Med	34	No	No	141
Birds	Class 10	20%	80%	25	Med	66.5	Yes	No	72
Soundscapes	Class 10	17%	83%	-	Med	41	No	No	117

**Table 8:** Machine learning classifiers performance with 34 signal features extracted from audio signals with 8 kHz sampling rate. Classifiers are built for 2 classes using 20-fold stratified cross-validation.

Classifier	Selected Parameter	Precision	Recall	F1	Accuracy
KNN	9	98.5	98.8	98.7	98.8
SVM with linear kernel	0.01	99	99	99	99.1
SVM with RBF kernel	20	98.3	98.6	98.9	99
Random Forest	500	98.1	98.7	99	99.1
Grading boosting	500	99.5	99.2	99.3	99.4
Extra Trees	100	99.2	98.8	99	99.2

**Table 9:** Models’ file locations.

Model	Description	Location ^1^
Extra trees	Model with the scaling mean/std vectors and the feature extraction parameters	extratrees_Mosquito and extratrees_MosquitoMEANS
Gradient Boosting	Model with the scaling mean/std vectors and the feature extraction parameters	gradientboosting_Mosquito and gradientboosting_MosquitoMEANS
KNN	Model with the feature extraction parameters	knn_Mosquito
Random Forest	Model with the scaling mean/std vectors and the feature extraction parameters	randomforest_Mosquito and randomforest_MosquitoMEANS
SVM	Model with the scaling mean/std vectors and the feature extraction parameters	svm_Mosquito, svm_MosquitoMEANS, svm_rbf_Mosquito and svm_rbf_MosquitoMEANS
CNN	Float and quantized model in C format	Float_model.cc and Quantized.cc

^1^https://github.com/DinarteVasconcelos/Models, (accessed on 25 July 2022).

**Table 10:** Confusion matrix for 81 files.

N = 81 Samples	Silence	Unknown	Aedes	Culex
**Silence**	5	1	5	3
**Unknown**	1	13	0	0
**Aedes**	0	0	32	1
**Culex**	0	0	0	20
